# Improving Liaison Psychiatry Referral Efficiency: A Quality Improvement Project in Liaison Psychiatry Services

**DOI:** 10.1192/j.eurpsy.2026.11715

**Published:** 2026-07-31

**Authors:** A. Iordache, M. Keenan, A. Tarar, A. Landers, I. Pozerski

**Affiliations:** Psychiatry, University Hospital Waterford, Waterford, Ireland

## Abstract

**Introduction:**

Prior to intervention, psychiatry referrals were paper-based and team-specific, resulting in several recurring issues. These included difficulty contacting referrers for missing information, misplaced or lost referrals, and delays in processing due to physical forms sitting unattended. The use of separate forms for General Adult and Psychiatry of Old Age created further confusion, with inappropriate referrals commonly made to the wrong service due to unclear triage criteria. There was also no reliable way to confirm when a referral had been submitted or received.

**Objectives:**

To streamline the referral process by implementing a unified email-based system and assess its impact on referral accuracy, accountability, and response time.

**Methods:**

A quality improvement intervention was implemented to address six recurring problems: 1) Difficulty contacting the referrer to clarify missing or incomplete information, 2) Misplaced or lost referrals, 3) Incorrect referral forms used due to service-specific templates, 4) Unclear criteria for General Adult vs. Psychiatry of Old Age referrals, 5) No tracking or acknowledgment system, and 6) Duplicate referrals for follow-up under the same admission due to lack of visibility. A unified referral system using a single dedicated email address and merged referral form was introduced. We collected baseline data for one month pre-intervention and compared it to data from one month post-intervention. Metrics assessed included referral volumes and processing improvements.

**Results:**

Referrals remained stable overall (62 before, 64 after), with significant process improvements noted. Referrals seen within 24 hours rose from 29 to 35, and those exceeding 72 hours dropped from four to zero. Although a few referrals were submitted without attachments, this was resolved via email follow-up. Average response time improved from 30.6 to 24.4 hours. Table 1 summarizes referral volumes; Table 2 outlines measurable process improvements. Figure 1 displays the response time shift.

**Image 1:**
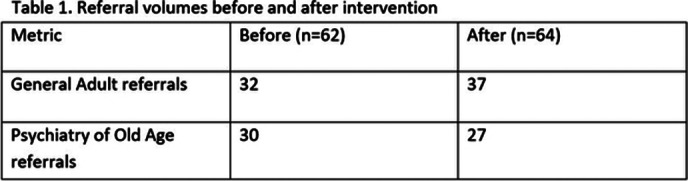

**Image 2:**
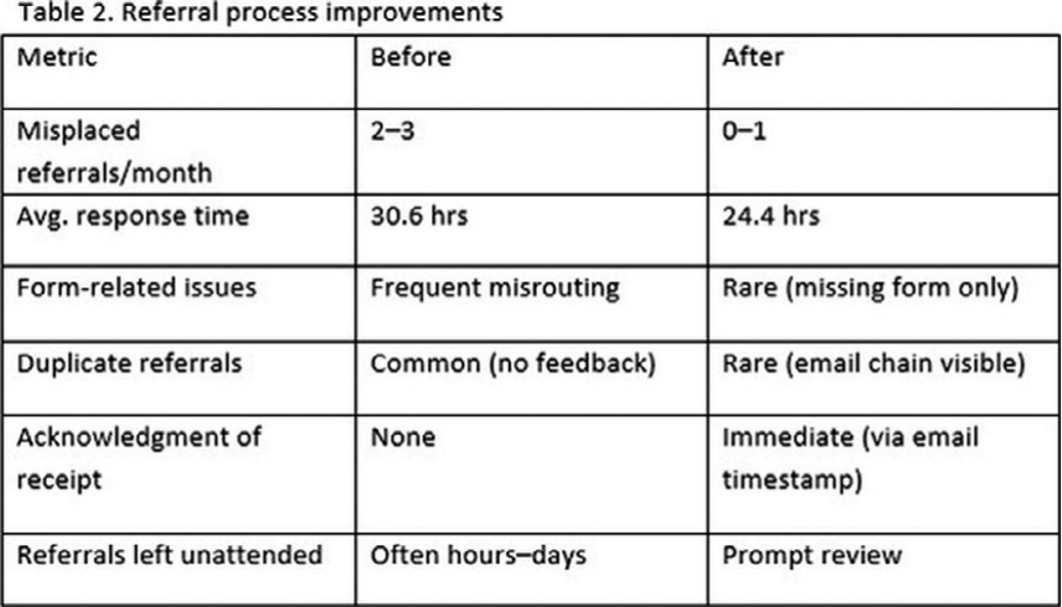
Image 2: Long description.

**Image 3:**
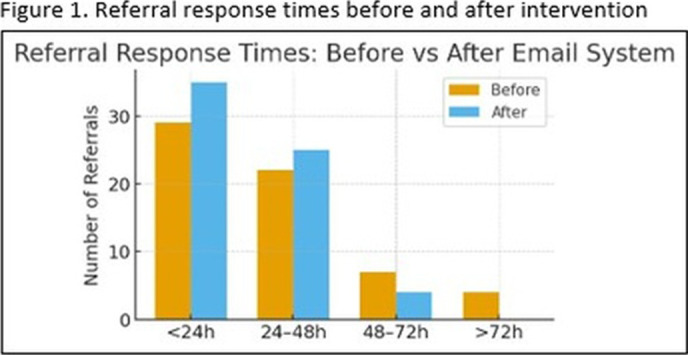
Image 3: Long description.

**Conclusions:**

The introduction of a unified email referral system led to measurable improvements in the efficiency, safety, and reliability of liaison psychiatry referrals. It significantly reduced delays, eliminated duplication, improved triage accuracy, and ensured timely communication with referrers. This simple, low-cost intervention enhanced both transparency and accountability in clinical workflows and represents a scalable model for similar improvements across other liaison psychiatry and mental health services.

**Disclosure of Interest:**

None Declared

## Long descriptions

### Image 2: Long description

The table consists of three columns: Metric, Before, and After.

* Misplaced referrals/month: decreased from 2–3 to 0–1.

* Avg. response time: decreased from 30.6 hrs to 24.4 hrs.

* Form-related issues: changed from Frequent misrouting to Rare (missing form only).

* Duplicate referrals: changed from Common (no feedback) to Rare (email chain visible).

* Acknowledgment of receipt: changed from None to Immediate (via email timestamp).

* Referrals left unattended: changed from Often hours–days to Prompt review.

Navigate back to Image 2:.

### Image 3: Long description

The y-axis is labeled Number of Referrals with a scale from 0 to 30 in increments of 10. The x-axis represents response time intervals. A legend in the top right identifies orange bars as Before and blue bars as After.

* For the less than 24h interval, the Before bar is at approximately 29 and the After bar increases to approximately 35.

* For the 24-48h interval, the Before bar is at 22 and the After bar increases to 25.

* For the 48-72h interval, the Before bar is at 7 and the After bar decreases to 4.

* For the greater than 72h interval, the Before bar is at 4 and there is no visible After bar, indicating a value of 0.

The data shows a shift toward faster response times following the email system implementation.

Navigate back to Image 3:.

